# Does inhaled nitric oxide treatment of pulmonary hypertension in extremely premature infants improve patient outcomes?

**DOI:** 10.1038/s41372-026-02666-1

**Published:** 2026-04-09

**Authors:** Tyler L. King, Patrick J. McNamara, Adrianne R. Bischoff

**Affiliations:** 1 Department of Pediatrics, Division of Neonatology, University of Iowa, Iowa City, IA USA; 2 Department of Internal Medicine, University of Iowa, Iowa City, IA USA

**Keywords:** Paediatrics, Respiratory tract diseases, Respiration

## Manuscript citation

Mirza H, Garcia J, Zussman M, Wadhawan R, Pepe J, Oh W. Inhaled Nitric Oxide Treatment of Early Pulmonary Hypertension to Reduce the Risk of Death or Bronchopulmonary Dysplasia in Infants Born Extremely Preterm: A Masked Randomized Controlled Trial. J Pediatr. 2025 Mar;278:114427. 10.1016/j.jpeds.2024.114427. Epub 2024 Dec 4. PMID: 39643111 [1].

## Question

Does inhaled nitric oxide (iNO) treatment for early echocardiographic pulmonary hypertension (PH) decrease the risk of death or bronchopulmonary dysplasia (BPD) in extremely preterm infants?

## Methods

### Design

Single-center double-blinded randomized control trial (RCT).

### Patients

Infants born less than or equal to 29 weeks’ gestation who received positive pressure ventilation at 72 ± 24 postnatal hours were eligible.

### Inclusion criteria

Screening echocardiograms were obtained at 72 ± 24 postnatal hours. PH was defined according to patent ductus arteriosus (PDA) gradient, tricuspid regurgitant peak velocity (TRV_max_), or end-systolic interventricular septal positioning.

### Exclusion criteria

Echocardiography exclusion criteria included flow associated PH from a large left to right PDA, severe left ventricular (LV) dysfunction (ejection fraction less than 40%) and/or presence of a complex cardiac defect. Patients were also excluded for prior iNO use, genetic conditions, major anomalies, or maternal COVID history.

### Randomization

Enrolled infants were categorized with moderate or severe PH. Moderate PH was characterized by an estimated pulmonary arterial pressure (PAP) greater than half to less than systemic systolic blood pressure (sBP) or flat end-systolic interventricular septum. Severe PH was characterized by estimated PAP equal or greater to systemic sBP or end-systolic septal bowing into the LV.

### Intervention

Patients were randomized to receive 20 ppm iNO or placebo. Randomization was stratified by gestational age and PH severity, irrespective of oxygen requirement. Intervention response was measured daily for 72 h, then every 24–48 h up to 14 days. Response was qualified by changes in PAP on echocardiogram and/or respiratory response through respiratory severity score (RSS), SpO_2_ / FiO_2_ ratio, or oxygenation index (OI). Interventions were weaned as PH resolved on echocardiogram or if patients were non-responders after 72 h of intervention. Non-responders were characterized by a lack of improvement in PAP on serial echocardiograms and/or less than 10% improvement from baseline RSS, SpO_2_ / FiO_2_ ratio, or OI. Intervention weaning occurred over 24 h and was suspended for 24 h for rebound PH or 10% absolute increase in supplemental oxygen.

### Primary outcome

The primary outcome was death or BPD at 36 weeks postmenstrual age. BPD was defined as requiring oxygen after a room air challenge or needing positive pressure ventilation.

### Statistical analysis

Statistical analysis for categorical variables and continuous variables were performed by Chi-square test or a Student’s *t* test/non-parametric tests, respectively. Relative risk was determined for the primary outcome.

## Results

### Patients

In total, 683 infants were screened; 180 patients were enrolled. Of these patients, 148 infants were excluded due to evidence of PH from a large left to right PDA, complex cardiac defects, or had prior iNO use. In total, 32 patients were randomized: 16 in each intervention arm, stratified by gestational age and PH severity. All, but one, infants were invasively ventilated and received surfactant. Two infants were categorized as severe PH, while the remaining had moderate PH. There were no differences in maternal or neonatal characteristics.

### Primary outcome

There was no difference in the primary outcome of death or BPD (68% iNO group vs 62% placebo, *p* = 0.99). Treatment response within 72 h of therapy did not differ (68% iNO group vs 93% placebo, *p* = 0.17)

### Safety outcome

Treatment duration did not differ between groups (median iNO therapy 99 h vs. 75 h placebo, *p* = 0.54). There were no differences in Grade 3 or 4 IVH (12.5% iNO vs. 12.5% placebo, *p* = 0.99), methemoglobinemia (*p* = 0.68), necrotizing enterocolitis (*p* = 0.23), LV dysfunction (*p* = 0.99), PDA requiring treatment (*p* = 0.99), hyperbilirubinemia (*p* = 0.99), or thrombocytopenia (*p* = 0.6) between groups.

### Additional outcomes

In total, 51 excluded infants received iNO clinically before enrollment and were more likely to have: lower gestational age (25.2 weeks vs. study cohort 26.2 weeks, *p* = 0.04), less antenatal steroids (65% vs. study cohort 94%, *p* < 0.01), lower 5 min APGARs (25% vs. study cohort 6%, *p* = 0.02), >60% FiO_2_ to maintain SpO_2_ > 85% for hypoxemic respiratory failure (HRF) (100% vs. study cohort 6%, *p* < 0.01), severe PH (29% vs. study cohort 6%, *p* = 0.01), any IVH (88% vs. study cohort 50%, *p* < 0.01) and death (47% vs. study cohort 6%, *p* < 0.01).

## Conclusion

The study ended at mid-interval data analysis. The authors concluded that iNO treatment for early PH without HRF may be futile. Similar treatment duration and responses were seen between intervention groups.

## Commentary

This single-center double-blinded RCT evaluated whether treatment of early echocardiographic PH with iNO would reduce BPD and mortality outcomes at 36 weeks’ postmenstrual age [1]. The study was terminated early, with the authors concluding that iNO treatment for early PH without HRF may be futile. The use of iNO in premature infants remains controversial. Historically, consensus guidelines from the National Institutes of Health (NIH) and American Academy of Pediatrics have discouraged iNO use in premature infants [2–4]. Yet, clinicians continue prescribing iNO based on clinical evidence of HRF or echocardiographic evidence of moderate to severe PH. These historical guidelines are limited by the lack of rigor in the adjudication of PH. Specifically, most trials only assessed for clinical markers of PH, primarily oxygenation index and respiratory severity. Use of echocardiography to characterize treatment response was not standardized. More recently, the NIH, American Heart Association, and American Thoracic Society report potential benefit of iNO use for premature infants with pulmonary arterial hypertension (resistance-mediated PH) [5, 6]. Observational studies where resistance-mediated PH is adjudicated by comprehensive echocardiography report iNO benefit, particularly in the transitional period [7, 8]. A recent prospective cohort study reported that infants as immature as 22 weeks’ gestation have an iNO response rate of 71% in early PH [9]. The discordance between clinical experience and RCT results requires thoughtful consideration.

The trial concluded there were no differences in BPD and mortality outcomes, with similar treatment response between intervention groups. However, this conclusion is subject to at least four major limitations.

First, the criteria used to assess PH response were limited to echocardiographic changes. Although the enrolled population satisfied the echocardiography criteria for moderate PH, the FiO_2_ at enrollment was less than 30%. The low baseline FiO_2_, unrepresentative of typical iNO use, likely limited detection of meaningful oxygenation changes, suggesting similar iNO response to placebo. Moreover, infants who received iNO treatment for HRF pre-enrollment were excluded, a group most likely to benefit. Exclusion of infants with more severe PH and worse clinical outcomes (increased IVH and mortality), are the likely population of interest and require prospective investigation.

Second, the dynamic nature of cardiopulmonary physiology and specific echocardiography parameters over time were not considered, which are critical in PH diagnosis and monitoring. Measurements to quantify PAP were used including PDA gradient, TRV_max_ and septal flattening but do not characterize different PH phenotypes. PH can be resistance-mediated, flow-mediated (i.e., hemodynamically significant PDA increasing pulmonary blood flow) and/or post-capillary (i.e., LV dysfunction). Although patients with flow-mediated (large left to right PDA shunts) and post-capillary (severe LV dysfunction) PH phenotypes were excluded at enrollment, the authors assumed a static PH phenotype for the enrolled population throughout their course. Importantly, iNO responders may transition from a resistance-mediated PH to a flow-mediated PH phenotype. Failure to recognize this transition and the need to wean iNO may lead to morbidity and mortality from unregulated augmentation of pre-ductal cardiac output (risk of reperfusion IVH) and post-ductal systemic hypoperfusion [10]. In summary, routine echocardiographic evaluation of PAP oversimplifies PH, hinders phenotype characterization, and may lead to therapeutic imprecision. These can be overcome by using a multiparametric assessment, as recommended by recent guidelines for targeted neonatal echocardiography [11].

Third, data on optimal iNO dosing and weaning in premature infants with resistance-mediated PH are limited. iNO dose and duration of exposure may be important modifiers of effect in extremely preterm infants, with some centers electing to start lower iNO doses with close monitoring and weaning as tolerated [10].

Fourth, in this study, PDA physiology may play a significant role when assessing iNO response to placebo. The authors did not address how PDA size and shunt direction impacted PH assessment, potentially overlooking subtle physiological changes among patients, including ductal closure and increases in left-to-right ductal shunting over time.

Taken together, these limitations indicate that the findings of the study should be interpreted with caution. The results of this study should not be used to broadly inform practice related to iNO use in infants with only echocardiographic confirmed PH.

## EBM lesson: generalizability, transportability, and effect modification

This study highlights the importance of assessing generalizability, transportability, and effect modification when appraising RCT findings. Both generalizability and transportability pertain to the target population. In this study, the target population was infants born less than 29 weeks’ gestation on respiratory support at 72 postnatal hours with echocardiographic PH. Generalizability applies the study findings to broader patient populations and may have problems when the study’s population is a subset of the target population [12]. In this case, the target population were infants that had echocardiograms confirming PH. However, most of these patients did not have clinical and echocardiography findings of PH and were excluded during the recruitment phase. The patients with both clinical and echocardiography confirmed PH represent the population most likely to benefit from iNO therapy both in the acute and long-term phase. Thus, the findings from this study may lack generalizability.

Transportability refers to moving from the target population to similar but different populations [13]. This is particularly important when applying this study design to patients in a different neonatal intensive care unit setting. When considering unit practice variability both respiratory support practices and PDA mitigation strategies can greatly impact airway recruitment and pulmonary vascular resistance. These differences across centers could alter the rate, presentation, and timing of onset of PH in extremely preterm infants. Additionally, the implementation of early echocardiography to appropriately identify the specific PH phenotype is an important modifier that may not be accessible to certain centers. Implementing these practices at other centers without additional clinical evidence to support a PH diagnosis may contribute to resource utilization without clear patient benefit. Therefore, when transportability is applied to this study, even slight variations in practice may impact the conclusions made from this study to other centers [13].

Effect modification considers the effect that a treatment has on different subgroups within the population [14]. In this case, it would include variables that may influence treatment response to iNO. Though this is not explicitly stated in the study, use of intravenous dopamine for hypotensive patients, a vasoactive medication that may directly increase pulmonary vascular resistance, may also have a modulator role [15]. It is plausible that iNO use would likely have a differential effect in infants on a dopamine infusion compared to those who were not and therefore would be an effect modifier. When applying these principles, if various patient characteristics and concurrent treatments for infants enrolled in studies like this do not have effect modification, then these variables are irrelevant to the study. Both generalizability and transportability are only impacted if effect modification exists and are key considerations when applying study findings to broader patient populations such as extremely premature infants with clinical PH. It is imperative to consider the application of these principles when appraising study findings that may suggest futility for a potentially efficacious therapy such as iNO in the correct patient population.

## Data Availability

Data sharing not applicable to this article as no datasets were generated or analyzed during this study.
